# To know or not to know: archiving and the under-appreciated historical value of data

**DOI:** 10.1186/1476-4598-7-18

**Published:** 2008-02-11

**Authors:** David Covarrubias, Maurice Van Emburgh, Hassan R Naqvi, Christian Schmidt, Shawn Mathur

**Affiliations:** 1 Section of Molecular Genetics and Microbiology and Institute for Cellular and Molecular Biology, University of Texas, 1 University Station, A 5000, Austin, TX 78712, USA; 2 Molecular Cancer, Biomed Central Ltd., Middlesex House, 34-42 Cleveland Street, London W1T 4LB, UK

## Abstract

Surplus goods, produced by a community, allow individuals to dedicate their efforts to abstract problems, while enjoying the benefits of support from the community. In return, the community benefits from the intellectual work, say, efficiently producing goods or profound medical aid. In further elevating quality of life, we need to understand nature and biology on the most detailed level. Inevitably, research costs are increasing along with the need for more scientists to specialize their efforts. As a result, a vast amount of data and information is generated that needs to be archived and made openly accessible with the permission to re-use and re-distribute. With economies undergoing crises and prosperity in an almost cyclic manner, it seems that funding for science and technology follows a similar pattern. Another aspect to the problem of the loss of data is the human propensity, at the level of each individual researcher, to passively discard data in the course of daily life and through a career. In a typical laboratory, significant amounts of information is still stored on disks in file cabinets or on isolated computers, and is lost when a research group disbands. Being conscientious to one's data, to see that it reaches a place in which it can persist beyond the lifespan of any one individual requires responsibility on the part of its creator.

## Editorial

What is progress? In a plain way, progress is an advance over an existing level. To obtain a quantifiable resolution, it is necessary to have references and contexts. References and contexts may comprise a fair number of data and references themselves. Where to start and what to consider? A comprehensive answer, consequently, requires an infinite amount of data and corresponding contexts. Ergo, each datum and context must be verifiable referenced and analyzed. Inevitably, the definition of progress requires a well-organized archive. How much effort is directed towards archiving? Furthermore, are enough resources available for maintaining data and documents? How can data that is stored in defunct formats and only accessible by obsolete programs be viably maintained for historical research? Is there sufficient support by and benefit for a society associated with such an activity?

Reversing the point of view, is the stored information appreciated and accessible? Taking it further, do we know what we have lost over time? Can we afford to selectively archive what we are able to preserve for future generations? Things we may not appreciate at this moment, take as given or consider as not suitable for in-depth investigation, are nevertheless records that could be missed at some time in the future. Answering the basic question from the preceding paragraph, there can never be enough effort in preserving information.

It is now assumed, simply for the sake of the argument, that there is no conscious selection of contents of an universal archive; access to this archive is arbitrarily set to unrestricted. Given that the archive contains a large number of data, one could mine this treasure for avoidance of costly errors and/or synthesize existing hypotheses to benefit existing approaches. In essence, are cultural/historical/scientific lessons the true currency of an interconnected society?

## Conclusion

Society as a whole has to decide how much resources are allocated towards preserving existing records, let alone the problem of failure to extract legacy data (we suggest the term **legacy data extinction**) in a technical and philosophical approach. Society as a whole, research groups, and the individual, each, has responsibility to decide how much resources are allocated towards preserving existing records. The problem of maintaining viable legacy data and the challenge of the human element, each, bear on preventing legacy data extinction to future humanity, in a technical and philosophical approach.

## Competing interests

DC, MVE, HRN and SM declare that there are no competing interests. CS is deputy editor of *Molecular Cancer *and receives no remuneration for his efforts.

## Authors' contributions

CS drafted and finalized this paper. MVE discussed ideas with CS that ultimately resulted in this paper and provided insightful critique. DC and SM assisted in gathering background information.

